# ﻿First discovery of the ant genus *Eburopone* Borowiec, 2016 (Hymenoptera, Formicidae, Dorylinae) in the Oriental realm, with description of a new species from Vietnam

**DOI:** 10.3897/zookeys.1184.109702

**Published:** 2023-11-10

**Authors:** Aiki Yamada, Dai Dac Nguyen, Katsuyuki Eguchi

**Affiliations:** 1 Systematic Zoology Laboratory, Department of Biological Sciences, Graduate School of Science, Tokyo Metropolitan University, 1-1 Minami-Osawa, Hachioji, Tokyo, 192-0397, Japan Tokyo Metropolitan University Hachioji Japan; 2 Institute of Ecology and Biological Resources, Vietnam Academy of Science and Technology, 18 Hoang Quoc Viet Road, Cau Giay District, Hanoi, Vietnam Institute of Ecology and Biological Resources, Vietnam Academy of Science and Technology Hanoi Vietnam; 3 Department of International Health and Medical Anthropology, Institute of Tropical Medicine, Nagasaki University, 1-12-4 Sakamoto, Nagasaki, Nagasaki, 852-8523, Japan Nagasaki University Nagasaki Japan

**Keywords:** Central Highlands, disjunct distribution, Indochina, morphology, non-army ant dorylines, taxonomy, Tay Nguyen

## Abstract

The doryline ant genus *Eburopone* Borowiec, 2016 currently contains only one valid species, *E.wroughtoni* (Forel, 1910) from southern Africa, with a considerable number of undescribed species awaiting formal description in the Afrotropical and Malagasy regions. In the present paper, *Eburoponeeasoana***sp. nov.** is described based on workers and dealate queens from a colony series collected in an evergreen forest on the Dak Lak Plateau of Vietnam (Ea So Nature Reserve, Dak Lak Province). The worker of the new species is morphologically clearly distinguished from *E.wroughtoni* by the combination of following characteristics: i) frontal line distinct, extending a little beyond mid-length of cranium; ii) anterior (frontoclypeal) margins of torulo-posttorular complex not forming conspicuous lobes protruding over anterior clypeal margin in full-face view; iii) mandibles when closed in full-face view forming only a little space between anterior clypeal margin and mandibles; iv) promesonotal suture faint and inconspicuous; v) abdominal segment III in dorsal view distinctly wider than long, with lateral margins only feebly convex. This represents the first discovery of the genus *Eburopone* in the Oriental realm, revealing the disjunct distribution of the genus. A partial sequence of the mitochondrial COI gene (658 bp) is provided as a DNA barcode for the new species. A worker-based key to the doryline genera of the Oriental realm is also provided.

## ﻿Introduction

The subfamily Dorylinae (Hymenoptera: Formicidae) is a clade of predatory ants primarily occurring in the tropics and subtropics of the world, consisting of true army ants and their relatives (Borowiec 2016, 2019). The generic-level classification of dorylines was comprehensively revised by Borowiec (2016) based on molecular phylogenetic analysis and morphological reevaluation. Prior to the publication of Borowiec (2016), the generic-level classification of non-army ant dorylines had long been in a state of confusion since Brown (1975). In particular, morphologically distinct lineages that are currently classified into nine genera had been placed within the formerly polyphyletic genus *Cerapachys* Smith, 1857.

*Eburopone* Borowiec, 2016 was one of the two genera newly established by the reclassification of the former *Cerapachys* members in Borowiec (2016). This genus currently contains only one valid species, *Eburoponewroughtoni* (Forel, 1910) from southern Africa (Bolton 2023), with a considerable number of undescribed species awaiting formal description in the Afrotropical and Malagasy regions (Fisher and Bolton 2016; Borowiec 2016; AntWeb 2023). Workers of the *Eburopone* are unique among the dorylines in having an externally visible whitish patch near the posterior edge of abdominal sternite IV, which is presumed to be glandular (Borowiec 2016).

*Eburoponewroughtoni* was originally described as *Cerapachyswroughtoni* based on workers from South Africa. Forel later described E.w.var.rhodesiana (Forel, 1913) and *E.roberti* (Forel, 1914) from Zimbabwe and South Africa, respectively. The latter two names were synonymized under *C.wroughtoni* by Brown (1975), for which the genus *Eburopone* was established by Borowiec (2016). There are so far no confirmed records of the genus outside Africa and Madagascar. Although Collingwood et al. (2011) recorded *E.wroughtoni* (as *Cerapachys*) from the United Arab Emirates, this record is dubious, as the AntWeb specimen cited as *C.wroughtoni* in the paper does not belong to *Eburopone*, but to *Parasyscia* Emery, 1882 which was treated as a junior synonym of *Cerapachys* at the time (more confusingly, a specimen image of Malagasy *Eburopone* from AntWeb was cited as a *Cerapachys* representative in the same paper).

**Figure 1.**
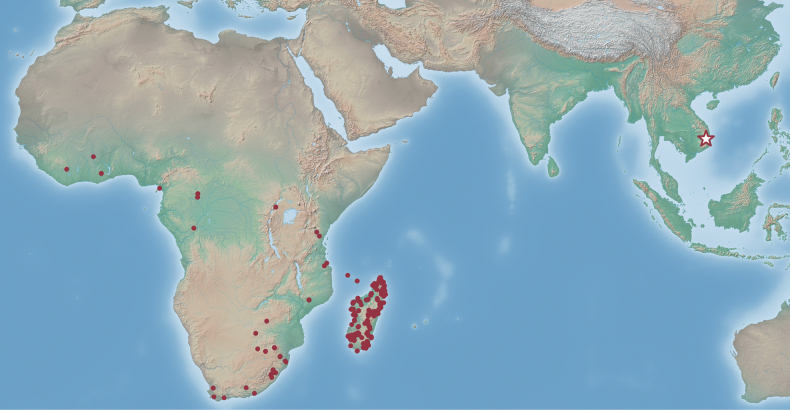
Distribution map of the genus *Eburopone*. The red dots represent locality data of *Eburopone* specimens (including undescribed species) available from AntWeb (2023); the star indicates the type locality of *E.easoana* sp. nov.

During our recent fieldwork in the Ea So Nature Reserve, Dak Lak Province, Central Highlands (Tay Nguyen) of Vietnam, we collected a colony fragment of an unknown doryline species. After careful morphological examination, this species is found to fit with the concept of *Eburopone* but is well distinguished from the only valid species *E.wroughtoni*. This represents the first discovery of the genus from the Oriental realm and reveals the disjunct distribution of the genus (Fig. 1), the geographic pattern of which is most reminiscent of the amblyoponine genus *Xymmer* Santschi, 1914 (Fisher and Bolton 2016; Satria et al. 2016; AntWeb 2023). We here describe the new species with some morphological remarks, based on the workers and dealate queens from the colony series. A partial sequence of the mitochondrial COI gene (658 bp) is also provided as a DNA barcode for the new species.

## ﻿Materials and methods

Abbreviations of the specimen depositories are as follows:

**IEBR** Institute of Ecology and Biological Resources, Vietnam Academy of Science and Technology, Hanoi, Vietnam;

**MCZC**Museum of Comparative Zoology, Cambridge, Massachusetts, USA;

**MHNG**Muséum d’Histoire Naturelle, Geneva, Switzerland;

**MNHAH**Museum of Nature and Human Activities, Hyogo, Japan;

**NHMUK**Natural History Museum, London, UK.

General morphological observations were made under epi-illumination using a Nikon SMZ1270 stereo microscope (Nikon, Tokyo, Japan) and a Nikon AZ100 multi-zoom microscope. Focus-stacked images of specimens under epi-illumination were produced by Affinity Photo 2.1.0 (Serif (Europe), Nottingham, UK) based on source images taken using a Nikon Z50 digital camera attached to the latter microscope via a NY-1S camera adapter (Micronet, Saitama, Japan). The retouching and adjusting functions of the software were used to improve the quality and clarity of stacked images.

Two workers from the same colony as the type series were used for further observations of some body parts by scanning electron microscope (SEM) JEOL JSM-6510 (JEOL, Tokyo, Japan) and transmission light microscope (TLM) Nikon Eclipse E600 (one of the specimens was also used for DNA extraction before conducting SEM and TLM observations). SEM was used to observe some morphological details of the cranium, promesonotal suture, and pronotomesopleural junction: the focal body parts were dissected and coated with platinum using a spatter coater JEOL JFC-1600 before SEM observation. TLM was used to observe the mouthparts: the mouthparts were dissected after cleaning by the DNA extraction protocol with Proteinase K described below, and slide-mounted with Euparal. The partly focus stacked images of the mouthparts under TLM were produced using Helicon Focus Pro 8.2.2 (Helicon Soft, Kharkov, Ukraine) based on source images taken by a Nikon Z5 digital camera attached to the TLM via a NY-1S35 camera adapter (Micronet).

Fifteen (or sixteen for queens) linear morphometric characters were measured using ImageJ 1.53e (National Institutes of Health, USA, available at https://imagej.nih.gov/ij/) based on photographs taken using a Nikon Z5 digital camera attached to a Nikon SMZ1270 stereo microscope via a NY-1S35 camera adapter (Micronet), and then ten (or eleven for queens) indices were calculated. Definitions of the worker morphometric characters basically follow those in Hita Garcia et al. (2017).

**HL** Head Length: maximum length of cranium in full-face view, measured from the midpoint of the anterior clypeal margin to the midpoint of the posterior margin of cranium;

**HW** Head Width: maximum width of cranium in full-face view (excluding compound eyes);

**EL** Eye Length: maximum diameter of compound eye in lateral view (for queens);

**OL** Ocellus Length: maximum diameter of median ocellus (for queens);

**SL** Scape Length: maximum length of antennal scape excluding basal condylar bulb;

**WL** Weber’s Length of Mesosoma: maximum diagonal distance of mesosoma in lateral view, measured from the angle at which the pronotum meets the cervix to posteroventral angle of metapleuron;

**DML** Dorsal Mesosomal Length: length of mesosomal dorsum from midpoint of anterodorsal margin of pronotum to midpoint of dorsal margin of propodeal declivity in dorsal view;

**PH** Pronotal Height: maximum height of pronotum in lateral view (for workers);

**PW** Pronotal Width: maximum width of pronotum in dorsal view;

**MFL** Metafemur Length: maximum length of metafemur, measured in dorsal view;

**PTH** Petiolar Height: maximum height of the petiolar tergum (abdominal tergum II) in lateral view, excluding petiolar sternum;

**PTL** Petiolar Length: maximum length of petiole (abdominal segment II) in dorsal view, measured from anterodorsal corner to posterior margin of posterior petiolar peduncle;

**PTW** Petiolar Width: maximum width of petiole (abdominal segment II) in dorsal view;

**A3L** Abdominal Segment III Length: maximum length of abdominal segment III in dorsal view, excluding presclerites (helcium);

**A3W** Abdominal Segment III Width: maximum width of abdominal segment III in dorsal view;

**A4L** Abdominal Segment IV Length: maximum length of abdominal segment IV in dorsal view, excluding presclerites;

**A4W** Abdominal Segment IV Width: maximum width of abdominal segment IV in dorsal view;

**CI** Cephalic Index: HW / HL × 100;

**SI** Scape Index: SL / HL × 100;

**EI** Eye Index: EL / HL × 100 (for queens);

**OI** Ocellus Index: OL / HL × 100 (for queens);

**DMI** Dorsal Mesosoma Index: PW / WL × 100;

**DMI2** Dorsal Mesosoma Index 2: DML / WL × 100;

**LMI** Lateral Mesosoma Index: PH / WL × 100 (for workers);

**MFI** Metafemur Index: MFL / HW × 100;

**LPI** Lateral Petiole Index: PTL / PTH × 100;

**DPI** Dorsal Petiole Index: PTW / PTL × 100;

**DA3I** Dorsal Abdominal Segment III Index: A3W / A3L × 100;

**DA4I** Dorsal Abdominal Segment IV Index: A4W / A4L × 100.

The morphological terminology follows Richter et al. (2020, 2021) for head, Liu et al. (2019) for mesosoma, Lieberman et al. (2022) for metasoma, and Boudinot (2015) for queen’s flight-related mesosomal sclerites. Following morphological concepts and terms are applied from Keller (2011) and Borowiec (2016): “torulo-posttorular complex”, “lateroclypeal teeth”, and “parafrontal ridges”. The term suture is used in a strict sense to refer to a groove that marks the line of fusion of two originally separate sclerites (Snodgrass 1962); under the definition, “promesonotal suture” and “pronotomesopleural suture” can be present only if the focal sclerites are fused. For setation, the term setae is used to refer to normal hair-like deflectable structures; the term chaetae is used to refer to differentiated peg-like or conical seta structures (Boudinot et al. 2020; Lieberman et al. 2022).

A partial sequence of mitochondrial cytochrome c oxidase subunit I gene (COI, 658 bp of standard DNA barcoding region) was determined from a single worker from the same colony as the type series. Total DNA was extracted using the Chelex-TE-ProK protocol: the specimen was washed and dissected in TE buffer, incubated at 56 °C for 24 h with 105 μL of the extraction buffer which contained 100 μL of 10% Chelex-TE solution (Chelex-100 resin, 143-2832, Bio-Rad Laboratories, California, USA) and 5 μL of Proteinase K (QIAGEN, Venlo, Netherlands), and lastly heated at 99 °C for 10 min for inactivating Proteinase K in the extraction buffer. PCR was performed with the primers LCO-EG (TTTCAACAAATCACAAAGAYATYGG) and HCO-EG (TAAACTTCAGGRTGACCRAAAAATCA). The PCR contained 5 μL of KOD One PCR Master Mix (KMM-101, TOYOBO, Osaka, Japan), 3.9 μl of distilled water, 0.3 μL of 10 pmol/μL forward and reverse primers (final 0.3 μM), and 1 μL of the DNA extract. The PCR thermal cycling condition was 40 cycles of 10 s at 98 °C, 5 s at 48.5 °C, and 5 s at 68 °C. After confirming the PCR amplification on a 2.0% agarose gel, the amplified product was incubated at 37 °C for 4 min and 80 °C for 1 min with ExoSAP-IT Express (Applied Biosystems, California, USA) to remove any excess primers and nucleotides. The cycle sequencing reaction was run with SupreDye Cycle Sequencing Kit v. 3.1 (AdvancedSeq, California, USA). The sequencing reaction product was purified and concentrated by ethanol precipitation with sodium acetate, and the nucleotide sequence was determined using an automated sequencer 3730xl DNA Analyzer (Applied Biosystems). Sequence assembly was performed using ChromasPro 1.7.6 (Technelysium, Queensland, Australia).

## ﻿Taxonomy

### ﻿ Eburopone

Taxon classificationAnimaliaHymenopteraFormicidae

Borowiec, 2016

8EA041D4-D90B-5B9B-A1DF-D6FBAD100D63

Eburopone Borowiec, 2016: 124. Type-species by original designation and monotypy: Cerapachyswroughtoni Forel, 1910.

#### Notes.

For general morphological diagnosis and description of the genus, see Borowiec (2016). See also Morphological remarks under the account of the new species.

### ﻿ Eburopone easoana sp. nov.

Taxon classificationAnimaliaHymenopteraFormicidae

B0BFD658-7E11-57F6-9104-A2110758231D

https://zoobank.org/73CBF0E9-4D91-4EAD-952B-F324E03D370E

**Figure 2.**
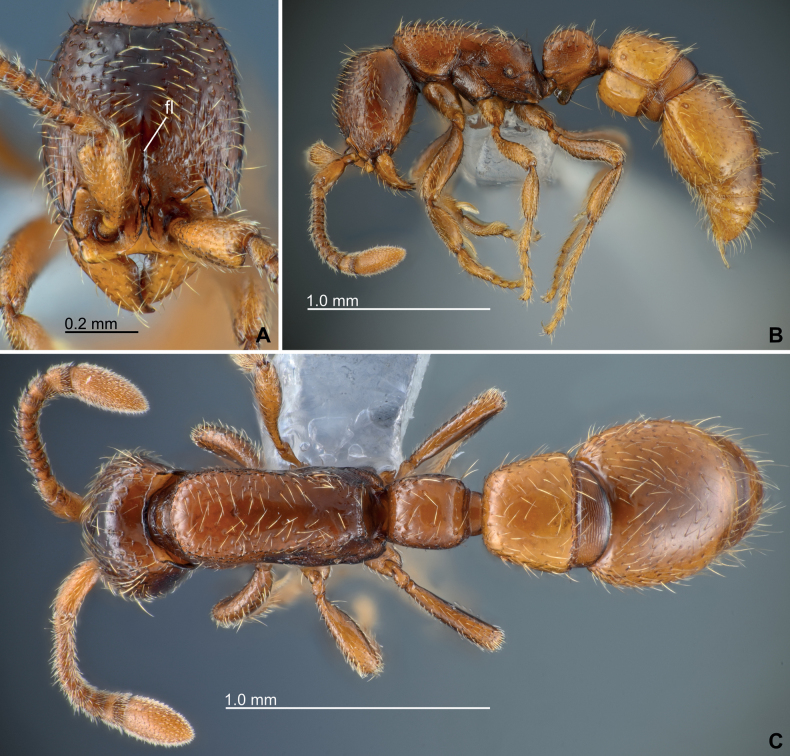
Habitus of *Eburoponeeasoana* sp. nov. worker, holotype, colony Eg17ix19-297 **A** head in full-face view **B** habitus in lateral view **C** habitus in dorsal view. Abbreviation: fl – frontal line.

**Figure 3.**
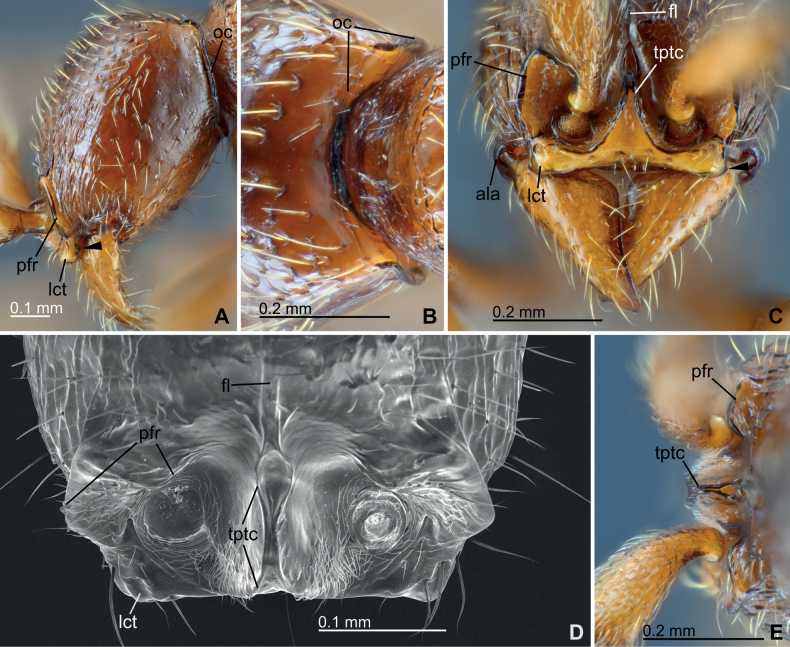
Cranium morphology of *Eburoponeeasoana* sp. nov. workers, colony Eg17ix19-297, holotype (**A–C, E**) and nontype (**D**) **A** head in lateral view **B** occipital corners in posterior view **C** anterior part of cranium in ventrofrontal view **D** scanning electron microscopic (SEM) photograph of anterior part of cranium in dorsofrontal view, antennae and mandibles removed (left antennal bulbus remains) **E** torulo-posttorular complex and parafrontal ridges in posterior vertical view against its posterior face. Black arrows in A and C indicate a weak protrusion on lateroclypeal teeth. Abbreviations: ala – atalar acetabulum; oc – occipital carina; fl – frontal line; lct – lateroclypeal teeth; pfr – parafrontal ridge; tptc – torulo-posttorular complex.

**Figure 4.**
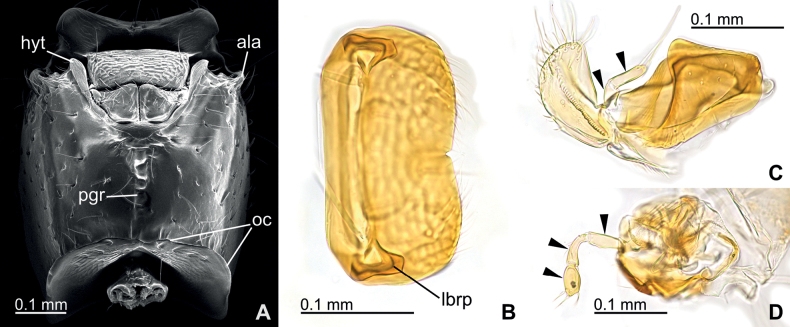
Cranial venter and mouthparts morphology of *Eburoponeeasoana* sp. nov. workers, colony Eg17ix19-297, nontypes **A** scanning electron microscopic (SEM) photograph of cranium in ventral view, mandibles removed **B** transmission light microscopic (TLM) photograph of labrum in frontal view, partly focus-stacked to emphasize lateral labral processes on caudal surface **C**TLM photograph of right maxilla in ventral view, with maxillary palpomeres indicated by black arrows **D**TLM photograph of labium in oblique lateral view, left side, with labial palpomeres indicated by black arrows. Abbreviations: oc – occipital carina; ala – atalar acetabulum; hyt – hypostomal teeth; lbrp – lateral labral process; pgr – postgenal ridge.

**Figure 5.**
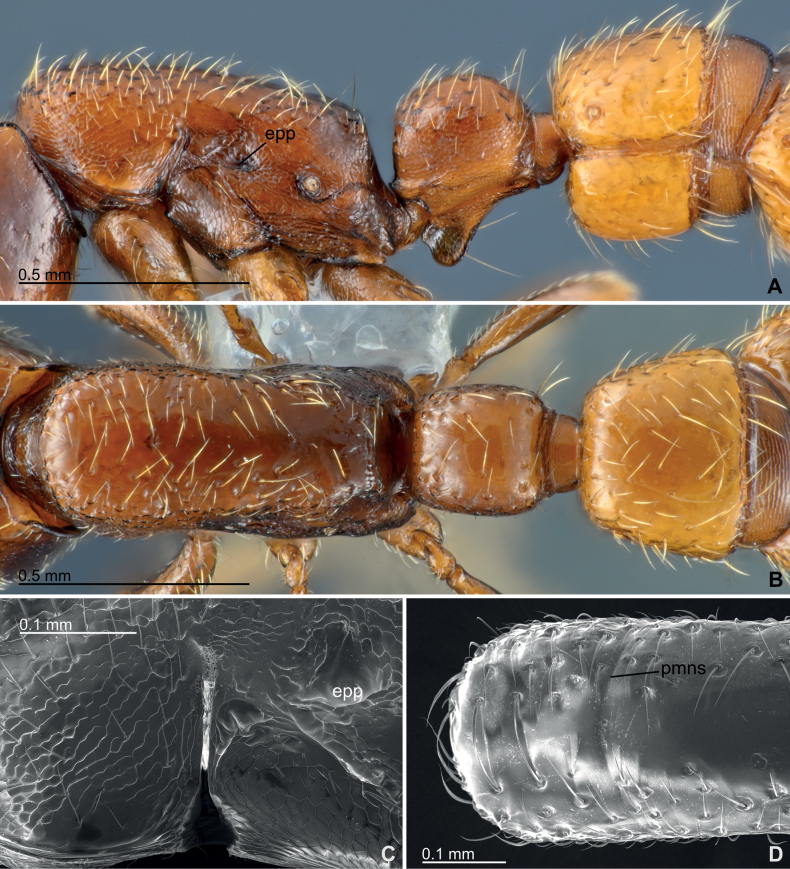
Mesosoma and abdominal segments II–III morphology of *Eburoponeeasoana* sp. nov. workers, colony Eg17ix19-297, holotype (**A, B**) and non-type (**C, D**) **A** mesosoma and abdominal segment II–III in lateral view **B** mesosoma and abdominal segment II–III in dorsal view **C** scanning electron microscopic (SEM) photograph of pronotomesopleural junction in lateral view **D**SEM photograph of promesonotum in dorsal view. Abbreviations: epp – pleural endophragmal pit; pmns – promesonotal suture.

**Figure 6.**
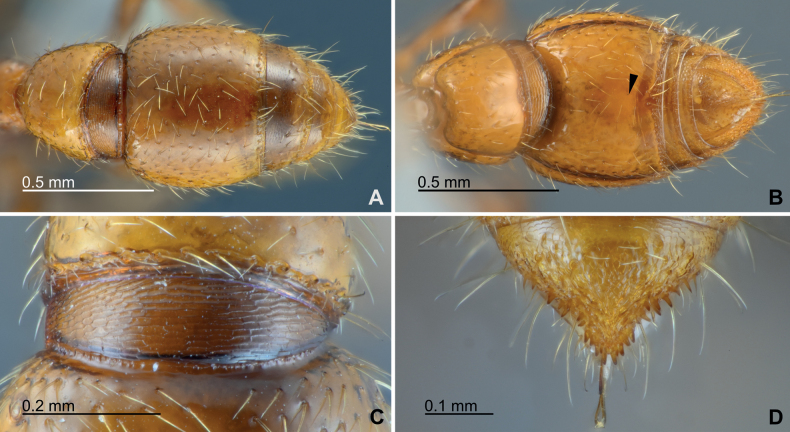
Abdominal segments IV–VII morphology of *Eburoponeeasoana* sp. nov. workers, colony Eg17ix19-297, holotype **A** abdominal segments IV–VII in dorsal view **B** abdominal segments IV–VII in ventral view, with the putative glandular patch of poststernite IV indicated by a black arrow **C** cinctus and presclerites of abdominal segment IV in dorsal view **D** abdominal tergite VII (pygidium) in dorsoposterior view.

**Figure 7.**
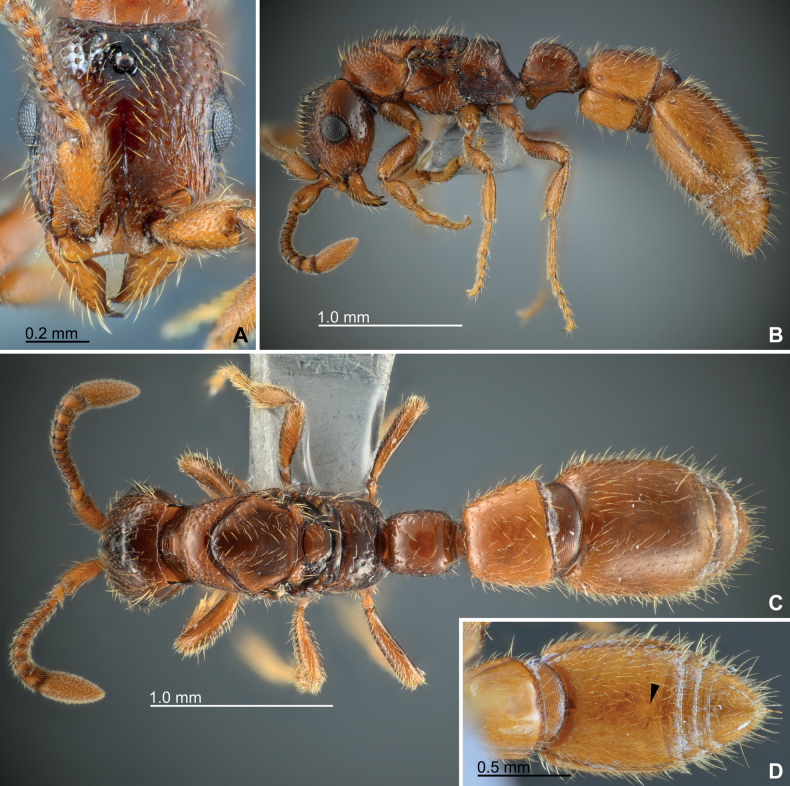
Habitus of dealate queens of *Eburoponeeasoana* sp. nov., colony Eg17ix19-297, paratypes (all except D are of the same individual) **A** head in full-face view **B** habitus in lateral view **C** habitus in dorsal view **D** abdominal segment IV–VII in ventral view, with the putative glandular patch of poststernite IV indicated by a black arrow.

Figs 2 , 3 , 4 , 5 , 6 , 7

#### Type material.

***Holotype*.** Vietnam • worker; Vietnam, Dak Lak Province, Ea Kar District, Ea So Nature Reserve; 12.9676°N, 108.5230°E, 478 m alt.; 17 Sept. 2019; K. Eguchi leg.; colony code Eg17ix19-297; IEBR.

***Paratypes*.** 6 workers and 2 dealate queens from the same colony as the holotype; IEBR, MCZC, MHNG, MNHAH.

#### Non-type material examined.

Two workers from the same colony as the type series, used for SEM and dissecting observations (one of these was used also for DNA barcoding).

#### DNA barcode.

A partial mitochondrial COI sequence of 658 bp length determined from a non-type worker (the same colony as the type series) is deposited at GenBank: accession number, LC776907.

#### Worker diagnosis.

Body rather bicolored: abdominal segments III–VII distinctively paler-colored than most surfaces of cranium and mesosoma. Frontal line distinct, extending a little beyond mid-length of cranium. Occipital corner in lateral view strongly produced posteriad to form conspicuous angle. Anterior (frontoclypeal) margins of torulo-posttorular complex not forming conspicuous lobes protruding over anterior clypeal margin in full-face view. Mandibles when closed in full-face view forming only a little space between anterior clypeal margin and mandibles. Promesonotal suture faint and inconspicuous. Petiole in dorsal view almost as long as or slightly longer than wide, with weakly convex lateral margins. Subpetiolar process in lateral view rounded lobate, with anterobasal margin weakly emarginate; posteroventral slope gentle, weakly concave. Abdominal segment III in dorsal view subtrapezoidal, strongly wider posteriorly, distinctly wider than long.

#### Worker measurements (in mm) and indices.

***Holotype***: HL 0.63, HW 0.55, SL 0.31, WL 0.86, DML 0.73, PH 0.31, PW 0.37, MFL 0.43, PTH 0.26, PTL 0.29, PTW 0.28, A3L 0.35, A3W 0.46, A4L 0.58, A4W 0.62, CI 87, SI 50, DMI 43, DMI2 85, LMI 36, MFI 79, LPI 111, DPI 97, DA3I 131, DA4I 107.

***Paratypes*** (*N* = 6): HL 0.63–0.65, HW 0.55–0.57, SL 0.31–0.33, WL 0.89–0.92, DML 0.74–0.80, PH 0.32–0.35, PW 0.37–0.40, MFL 0.44–0.47, PTH 0.27–0.29, PTL 0.30–0.33, PTW 0.29–0.32, A3L 0.37–0.43, A3W 0.47–0.51, A4L 0.56–0.64, A4W 0.64–0.66, CI 86–88, SI 48–51, DMI 42–43, DMI2 84–87, LMI 36–38, MFI 80–83, LPI 111–114, DPI 95–98, DA3I 118–130, DA4I 103–112.

#### Queen measurements (in mm) and indices.

***Paratypes*** (*N* = 2): HL 0.70–0.72, HW 0.58–0.61, EL 0.19–0.20, OL 0.05–0.06, SL 0.35–0.36, WL 1.18–1.20, DML 1.07–1.08, PW 0.57–0.58, MFL 0.51–0.53, PTH 0.31, PTL 0.35–0.36, PTW 0.35–0.37, A3L 0.48–0.49, A3W 0.56–0.58, A4L 0.82, A4W 0.73–0.75, CI 84, SI 49–50, EI 28, OI 7–8, DMI 48, DMI2 90–91, MFI 87–88, LPI 111–117, DPI 100–101, DA3I 115–119, DA4I 89–91.

#### Description.

**Worker. *Body coloration*.** Body rather bicolored: cranium, mesosoma, and petiole, mostly dark reddish brown; antennae, anterior part of cranium, mandibles, legs, and abdominal segments III–VII paler brownish to yellowish (Fig. 2A–C). Pleural endophragmal pit conspicuously blackish.

***Head structure*.** Cranium in full-face view subrectangular, 1.14–1.17× longer than wide (CI, 86–88), with convex lateral margins; posterior margin widely weakly concave. Occipital corner in lateral view strongly produced posteriad to form conspicuous angle. Occipital carina (oc, Fig. 3A, B) distinct and completely encircles occiput, forming inconspicuous and slightly undulate carina dorsally and a conspicuous flange lateroventrally. Frontal line (fl, Figs 2A, 3C, D) distinct, extending a little beyond mid-length of cranium. Torulo-posttorular complex (tptc, Fig. 3C–E) in full-face view arrow-like shaped with narrowed median part; anterior (frontoclypeal) margins not forming conspicuous lobes protruding over anterior clypeal margin in full-face view; maximal width of posterior arrowhead-like part a little shorter than major diameter of antennal socket. Anterior clypeal margin evenly weakly concave in full-face view. Lateroclypeal teeth (lct, Fig. 3A, C, D) large, rounded lobate, strongly protruding latero-anteriad to form anteriormost points of cranium, lateroventrally with a weak protrusion (black arrows in Fig. 3A, C). Parafrontal ridges (pfr, Fig. 3A, C–E) conspicuous and strongly elevated, turning at posteriorly to incompletely surround antennal sockets, and forming well-marginated subtriangular mound-like lobes; its lateral margins evenly rounded arc-like in full-face view; minimal distance from medialmost end of parafrontal ridge to margin of torulo-posttorular complex in full-face view as long as major diameter of antennal socket. Eye and ocelli completely absent. Antenna 12-merous; scape short, just reaching approximately mid-length of cranium when laid backward; antennomere XII distinctively longer than summed length of three preceding antennomeres IX–XI. Mandible subtriangular, with rounded obtuse basal angle and blunt apex, when closed in full-face view forming only a little space between anterior clypeal margin and mandibles (basal angles nearly reaching center line of cranium when mandibles closed); masticatory margin virtually edentate but with a series of feeble inconspicuous denticles. Postgenal ridge (pgr, Fig. 4A) externally recognizable as a broad cross-ribbed furrow, ending a little before the level of occipital carina. Hypostomal teeth (hyt, Fig. 4A) large and conspicuous with rounded apex in ventral view. Atalar acetabulum (ala, Figs 3C, 4A) strongly produced anterolaterad. Labrum subrectangular with distal margin weakly bilobed with a small median notch; lateral labral process (lbrp, Fig. 4B) not visible in frontal view, strongly produced distad and truncated tooth-like. Maxillary palps bi-merous; palpomere II elongate, much longer than palpomere I (Fig. 4C). Labial palps tri-merous; palpomere III distinctly shorter than preceding palpomeres; palpomere II strongly curved basally (Fig. 4D).

***Mesosomal structure*.** Mesosoma with evenly and slightly convex dorsum in lateral view (Fig. 5A); lateral margins in dorsal view almost parallel, weakly and widely compressed laterally around mesopleura (Fig. 5B). Pronotal flange only weakly marginated posteriorly from collar. Pronotum and mesopleuron unfused in terms of absence of sclerotized fusion, with dorsal part of pronotomesopleural junction apparently connected by a narrow intersegmental membrane (Fig. 5A, C). Promesonotal suture inconspicuous, only faintly recognizable as a shallow groove (pmns, Fig. 5D; may be hardly recognizable under normal optical observation as in Fig. 5B, but the weak suture can be recognizable by changing angle of observation or lighting condition). Concavity surrounding pleural endophragmal pit (epp, Fig. 5A, C) deep and conspicuous. Propodeal declivity only weakly marginate dorsally, almost immarginate laterally; dorsal margin weakly arched anteriad in dorsal view (Fig. 5A, B). Posterodorsal corner of propodeum in lateral view blunt, only weakly angulated. Propodeal lobe evenly rounded in lateral view. Pretarsal claws simple without teeth.

***Metasomal structure*.** Petiole (abdominal segment II) in dorsal view 1.02–1.05× longer than wide (length measured from anterodorsal corner to posterior margin of posterior petiolar peduncle; DPI, 95–98), with weakly convex lateral margins; posterior margin of posterior petiolar peduncle slightly arched anteriad (Fig. 5B). Dorsal outline of petiole in lateral view roundly convex; anterolateral petiolar carina dorsally protrude to form a stout prominence with acute apex. Subpetiolar process in lateral view rounded lobate, with anterobasal margin weakly emarginate; posteroventral slope gentle, weakly concave (Fig. 5A). Abdominal segment III in dorsal view subtrapezoidal, strongly wider posteriorly, 1.18–1.31× wider than long (DA3I, 118–131; Fig. 5B); lateral margins only feebly evenly convex; 1.59–1.62× wider than petiole. Abdominal posttergite III subrectangular in lateral view, with almost vertical anterior face; distinctly larger in height than fused poststernite III (Fig. 5A). Abdominal segment IV in dorsal view 1.03–1.12× wider than long (DA4I, 103–112), with strongly convex lateral margins (Fig. 6A). Putative glandular patch near posterior edge of abdominal poststernite IV feebly recognizable as oval pale area contrasted by little darker-colored proximate surrounding (black arrow in Fig. 6B; no specialized structure such as gland opening recognizable on the external sternal surface).

***Setation and sculpture*.** Body setation relatively sparse. Anterolateral surfaces of cranium coarsely longitudinally rugose (Fig. 2A); other areas of cranium basically smooth except having sparse seta-bearing foveae; posterolateral surfaces very smooth with few setae and sculpturing (Fig. 3A). Frontal face of labrum reticulate (Fig. 4A). Proximal face of maxillary stipes mostly smooth. Dorsum of mesosoma and petiole smooth and shiny with sparse seta-bearing foveae; pronotal flange, lateral surfaces of mesosoma and petiole shiny but superficially reticulate-imbricate (Fig. 5A–C); propodeal declivity and anterior face of petiole smooth. Abdominal postsclerites III and IV smooth, except sparse seta-bearing foveae. Cinctus of abdominal segment IV almost smooth, with coarse short longitudinal ribs near its anterior margin (Fig. 6C). Presclerites of abdominal segment IV reticulate-imbricate. Abdominal tergites V–VI largely smooth with superficially imbricate anterior face. Abdominal tergite VII (pygidium) coarsely areolate-reticulate except superficially imbricate anterior face, armed with numerous stout chaetae arranged in two or three irregular oblique-longitudinal rows along each posterolateral margin (Fig. 6D).

**Dealate queen.** Largely similar to the worker except for some queen-specific features. Eyes and ocelli large and conspicuous (Fig. 7A, B); eyes circular with ~ 20 ommatidia at maximum diameter in lateral view; minimal distance between margins of median and lateral ocellus as long as major diameter of median ocellus; minimal distance between margins of lateral ocelli ~ 2× major diameter of median ocellus. Mesosoma with fully developed flight sclerites and wing basal remnants (Fig. 7C); pronotum dorsolaterally coarsely foveolate-reticulate; notauli weakly present as coarse groove; parapsidal lines distinct; mesopleuron almost smooth; oblique mesopleural sulcus deep and conspicuous. Petiole in dorsal view almost as long as wide (DPI, 100–101); abdominal segment III in dorsal view 1.15–1.19× wider than long (DA3I, 115–119); abdominal segment IV in dorsal view 1.10–1.12× longer than wide (DA4I, 89–91). Putative glandular patch near posterior edge of abdominal poststernite IV feebly recognizable as in worker (black arrow in Fig. 7D).

**Male.** Unknown.

#### Etymology.

The specific epithet is named after the type locality, Ea So Nature Reserve: *easo* combined with the Latin feminine suffix -*ana*, adjective.

#### Habitat.

The collecting site of the type colony series is primarily covered with a relatively disturbed secondary evergreen forest. The collector (K. Eguchi) did not record the exact microhabitat and collecting situation of the colony fragment.

#### Differences from *E.wroughtoni*.

The worker of *E.easoana* is morphologically easily distinguished from the only other valid congener *E.wroughtoni* from southern Africa (see also the account of *E.wroughtoni* below) by the combination of following characteristics: i) frontal line distinct, extending a little beyond mid-length of cranium; ii) anterior (frontoclypeal) margins of torulo-posttorular complex not forming conspicuous lobes protruding over anterior clypeal margin in full-face view (anterior clypeal margin evenly weakly concave in full-face view); iii) mandibles when closed in full-face view forming only a little space between anterior clypeal margin and mandibles (basal angles nearly reaching center line of cranium when mandibles closed); iv) promesonotal suture faint and inconspicuous; v) abdominal segment III in dorsal view distinctly wider than long, with lateral margins only feebly convex.

#### Morphological remarks.

The morphology of workers of *E.easoana* is generally consistent with the concept of *Eburopone* in Borowiec (2016), except for the number of labial palpomeres. Borowiec (2016) stated that both the maxillary and labial palps of *Eburopone* are bi-merous in workers. However, *E.easoana* has a bi-merous maxillary and tri-merous labial palps (Fig. 4C, D), indicating the inter-specific variation of the character within the genus. According to Borowiec (2016), in dorylines, the labial palps of workers have, as a rule, fewer palpomeres than the maxillary palps of the same individual, except for the New World army ants and *Acanthostichus* Mayr, 1887. Therefore, *E.easoana* represents a new exception against this rule. The known palp formula of *Eburopone* worker is updated to 2, 2 or 2, 3.

Borowiec (2016) described the condition of pronotomesopleural junction in *Eburopone* workers “pronotomesopleural suture visible as groove but not unfused”. However, the pronotomesopleural junction of the *E.easoana* workers can be interpretable as unfused in terms of the absence of sclerotized fusion, since the “groove” between the two sclerites seems membranous (Fig. 5A, C). Based on examination of the specimen images on AntWeb, the pronotomesopleural junction of *E.wroughtoni* also seems to be in the same condition.

### ﻿ Eburopone wroughtoni

Taxon classificationAnimaliaHymenopteraFormicidae

(Forel, 1910)

ECBB48E0-6572-5887-BCEC-448545BBF690

Cerapachys wroughtoni Forel, 1910: 422. Emery 1911: 9; Arnold 1915: 16; Wheeler 1922: 756; Brown 1975: 24, 63; Bolton 1995: 145; Collingwood et al. 2011 (dubious record): 410; Borowiec 2014: 62. Combination in C. (Cerapachys): Emery 1911: 99. Combination in Eburopone: Borowiec 2016: 129.
Cerapachys wroughtoni var. rhodesiana Forel, 1913: 212. Arnold 1915: 16; Wheeler 1922: 756. Junior synonym of C.wroughtoni: Brown 1975: 24; Bolton 1995: 144.
Cerapachys roberti Forel, 1914: 212. Wheeler 1922: 756; Arnold 1926: 192. Junior synonym of C.wroughtoni: Brown 1975: 24; Bolton 1995: 144.

#### Type material images examined.

The following type materials of *E.wroughtoni* and its two junior synonyms were non-physically examined based on the images available from AntWeb (https://www.antweb.org): *E.wroughtoni* (Forel, 1910), syntype, CASENT0249876 (MHNG); E.wroughtonivar.rhodesiana (Forel, 1913), syntype, CASENT0907055 (MHNG); *E.roberti* (Forel, 1910), syntypes, CASENT0907054 (MHNG), CASENT0902707 (NHMUK).

#### Notes.

Although Brown (1975) treated two southern African *Eburopone* (sub)species, *E.roberti* and *E.w.rhodesiana* as junior synonyms of *E.wroughtoni*, the types of these names show conspicuous morphological differences, especially between *E.w.rhodesiana* and the others. *Eburoponew.rhodesiana* is the most morphologically distinct among these by i) lateroventral flange of occipital carina less developed; ii) promesonotal suture inconspicuous (hardly recognizable in the examined image); iii) abdominal segment III distinctly wider than long. *Eburoponeroberti* is different from the syntype of *E.wroughtoni* at least by having deeper and more prominent promesonotal suture. The differences suggest a possible heterospecificity of these names, especially for *E.w.rhodesiana*. However, their taxonomic statuses are not addressed in the present study, since it would be appropriate to examine additional colony series; additionally, many undescribed species are known from the Afrotropical and Malagasy regions. Even if multiple species are confused under *E.wroughtoni*, the independence of *E.easoana* at the species level is unquestionable.

### ﻿Worker-based key to doryline genera of the Oriental realm

The following key is based on the generic descriptions and global key to genera of Dorylinae by Borowiec (2016).

**Table:** 

1	Abdominal tergite VII (last visible tergite, pygidium), not armed with numerous differentiated stout chaetae (“peg-like setae”), at most with only a few pairs of cuticular projections	**2**
–	Abdominal tergite VII armed with numerous stout chaetae	**3**
2	Abdominal segment III forms differentiated “postpetiole”, narrowly attached to segment IV	***Aenictus* Shuckard, 1840**
–	Abdominal segment III not differentiated and broadly attached to segment IV	***Dorylus* Fabricius, 1793**
3	Abdominal segment III broadly attached to segment IV, without a conspicuous anterior constriction of segment IV	***Yunodorylus* Xu, 2000**
–	Abdominal segment III at least weakly differentiated from segment IV, with a conspicuous anterior constriction of segment IV	**4**
4	Meso- and metatibiae each with two spurs; most body surface with prominent costate sculpture	***Chrysapace* Crawley, 1924**
–	Mesotibiae with a single spur or without spurs and metatibiae always with a single spur	**5**
5	Mesotibiae without spurs	***Simopone* Forel, 1891**
–	Mesotibiae with a single spur	**6**
6	Petiole (abdominal segment II) dorsolaterally marginate at least in anterior portion; metacoxae usually with a conspicuously lamellate posterior flange	***Lioponera* Mayr, 1879**
–	Petiole (abdominal segment II) not conspicuously dorsolaterally marginate; if petiole appearing marginate, metacoxae without a conspicuously lamellate posterior flange	**7**
7	Pronotum and mesopleuron completely fused, never with a deep cut of pronotomesopleural junction	**8**
–	Pronotum and mesopleuron unfused, with a deep cut of pronotomesopleural junction	**9**
8	Constrictions present at anterior end of abdominal segments V and VI	***Zasphinctus* Wheeler, 1918**
–	Constrictions absent at anterior end of abdominal segments V and VI	***Parasyscia* Emery, 1882**
9	Constrictions present at anterior end of abdominal segments V and VI	***Eusphinctus* Emery, 1893**
–	Constrictions absent at anterior end of abdominal segments V and VI	**10**
10	Antennae 12-merous	**11**
–	Antennae 8- to 11-merous	**12**
11	Eyes conspicuous with more than 20 ommatidia; abdominal sternite IV normal without a whitish patch near the posterior edge	***Cerapachys* Smith, 1857**
–	Eyes absent or perhaps vestigial with at most several weakly differentiated ommatidia; abdominal sternite IV with a unique whitish patch near the posterior edge (may be feeble)	***Eburopone* Borowiec, 2016**
12	Abdominal tergite IV in lateral view folding over sternite IV and the anterior portion of sternite at least partly obscured; abdominal segment III relatively wide in dorsal view and larger than the preceding abdominal segment II (petiole)	***Syscia* Roger, 1861**
–	Abdominal tergite IV not folding over sternite IV and the anterior portion of the sternite visible; abdominal segment III relatively narrow in dorsal view and similar in size to the preceding abdominal segment II (petiole)	***Ooceraea* Roger, 1862**

## Supplementary Material

XML Treatment for Eburopone

XML Treatment for Eburopone easoana

XML Treatment for Eburopone wroughtoni

## ﻿Additional information

### Author ORCIDs

Aiki Yamada https://orcid.org/0000-0002-2141-0661

Dai Dac Nguyen https://orcid.org/0009-0006-8024-2553

Katsuyuki Eguchi https://orcid.org/0000-0002-1054-1295

### Data availability

All of the data that support the findings of this study are available in the main text.
